# Planetary health: challenges and critical nursing action

**DOI:** 10.1590/0034-7167.2025780202

**Published:** 2025-06-30

**Authors:** Jacinta de Fatima Sena da Silva

**Affiliations:** I Fiocruz, Brasília. Professor of the Graduate Program in Public Health Policies, Angicos-Center for Popular Education in Health and Health, Environment and Work Program. Universidade de Brasília, Department of Public Health. Researcher and Professor. President of ABEn (2022-2025). Brasília, Federal District, Brazil.

The concept of planetary health^(1)^ encompasses the relationship between human well-being and the preservation of ecosystems. Therefore, intense climate change and environmental degradation present challenges for collective health that require innovative approaches and concrete actions. Nursing, historically committed to health care and promotion, has a strategic role in this process.

In line with this relevant theme, the Brazilian Nursing Association (in Portuguese, *Associação Brasileira de Enfermagem* - ABEn) proposed for the 86^th^ Brazilian Nursing Week (in Portuguese, *Semana Brasileira de Enfermagem* - SBEn) to bring to the center of the debate one of the most urgent themes of the present day: “Planetary health: challenges and nursing critical action”.

From this perspective, the 86^th^ SBEn seeks to strengthen the debate and mobilize professionals, students and researchers for a positive agenda based on sustainability, equity and social justice. More than just responding to the consequences of environmental and climate emergencies, nursing needs to actively position itself in the construction of public policies and the implementation of sustainable practices in its various fields of activity.

## Current debates in a historical and celebratory initiative

SBEn, held annually from May 12 to 20, is marked by the birth dates of Florence Nightingale and the death of Anna Nery. Created in 1940 by the director of *Escola de Enfermagem Anna Nery* and a historical figure of ABEn, Laís Netto dos Reys, SBEn is an initiative of ABEn to strengthen nursing in Brazil. Starting in 1958, with the support of state sections, it began to mobilize schools and institutions throughout the country. Officialized in 1960 by Juscelino Kubitschek, SBEn seeks to promote scientific, cultural and social meetings among professionals. Each year, ABEn reaffirms this political act that has become a tradition in the field.

Training and continuing education are essential pillars for preparing professionals who understand the complexity of environmental, climate and health challenges and are agents of change in the promotion of healthy and sustainable territories. Furthermore, the fight for decent working conditions and professional recognition is directly connected to the environmental agenda. The overexploitation of health work reflects the same model that exhausts natural resources and compromises life on the planet. Therefore, thinking about planetary health also means rethinking labor relations and the socioeconomic impacts that affect nursing and society as a whole.

Nursing has the responsibility and the opportunity to be at the forefront of developing proposals and practices, promoting care that considers not only human health, but planet integrity. Thus, the 86^th^ SBEn is structured around three central axes, which deepen this discussion:

**Axis 1. Nursing practices in multiple scenarios that promote planet health:** highlights the role of nursing workers in implementing sustainable practices, such as responsible management of resources in healthcare services and addressing the impacts of climate change in care settings.

**Axis 2. Transformative education as a path to preserving life in its various forms:** highlights the importance of education and literacy in planetary health, and the challenges for training and continuing education. It also addresses the multiple perspectives on the diverse environmental-territorial realities regarding teaching and its potential for transformation and expansion of knowledge and awareness, and also on the training process of nursing professionals with the social role of agents of transformation.

**Axis 3. Changes in the world of work and their impacts on planetary survival:** discusses the relationship between precarious work, exploitation of natural resources and the challenges for the valorization of nursing, highlighting the importance of public policies that combat overexploitation and promote decent working conditions.

By integrating the theme and its three axes, the 86^th^ SBEn highlights the need for nursing to be engaged in building a supportive and sustainable future, guided by ethics, social justice and commitment to life in all its forms.
