# Supplementary material for: Planetary health: challenges and critical nursing action
Source: Rev Bras Enferm. 2025 Jun 30;78(2):e780202. doi: 10.1590/0034-7167.2025780202 (PMC12221269; doi:10.1590/0034-7167.2025780202)
Supplement: Translation

# Translation: Salud planetaria: desafíos y acción crítica de enfermería

**DOI:** 10.1590/0034-7167.2025780202es

**Published:** 2025-05-16

**Authors:** Jacinta de Fatima Sena da Silva

**Affiliations:** I Fiocruz, Brasília. Profesor del Programa de Postgrado en Políticas Públicas de Salud, Angicos-Centro de Educación Popular en Salud y Programa de Salud, Ambiente y Trabajo. Universidade de Brasília, Departamento de Salud Pública. Investigador y Docente. Presidente de ABEn (Gestión 2022-2025). Brasília, Distrito Federal, Brasil.

El concepto de salud planetaria^(1)^ abarca la relación entre el bienestar humano y la preservación de los ecosistemas. Por lo tanto, el intenso cambio climático y la degradación ambiental plantean desafíos para la salud pública que requieren enfoques innovadores y acciones concretas. La enfermería, históricamente comprometida con el cuidado y la promoción de la salud, tiene un papel estratégico en este proceso.

En sintonía con esta temática relevante, la Asociación Brasileña de Enfermería (en portugués, *Associação Brasileira de Enfermagem* - ABEn) propuso para la 86ª Semana Brasileña de Enfermería (en portugués, *Semana Brasileira de Enfermagem* - SBEn) traer al centro del debate uno de los temas más urgentes de la actualidad: “Salud planetaria: desafíos y la acción crítica de la enfermería”.

Desde esta perspectiva, la 86ª SBEn busca fortalecer el debate y movilizar a profesionales, estudiantes e investigadores hacia una agenda positiva basada en la sostenibilidad, la equidad y la justicia social. Más que simplemente responder a las consecuencias de las emergencias ambientales y climáticas, la enfermería necesita posicionarse activamente en la construcción de políticas públicas y la implementación de prácticas sostenibles en sus diversos campos de actividad.

## Debates actuales en una iniciativa histórica y celebratoria

SBEn, que se celebra anualmente del 12 al 20 de mayo, está marcado por las fechas de nacimiento de Florence Nightingale y la muerte de Anna Nery. Creada en 1940 por la directora de la *Escola de Enfermagem Anna Nery* y figura histórica de la ABEn, Laís Netto dos Reys, la SBEn es una iniciativa de la ABEn para fortalecer la enfermería en Brasil. A partir de 1958, con el apoyo de secciones estatales, comenzó a movilizar escuelas e instituciones en todo el país. Fundada oficialmente en 1960 por Juscelino Kubitschek, SBEn busca promover encuentros científicos, culturales y sociales entre profesionales. Cada año, ABEn reafirma este acto político que se ha convertido en una tradición en el ámbito.

La formación y la educación continua son pilares esenciales para preparar profesionales que comprendan la complejidad de los desafíos ambientales, climáticos y de salud, y sean agentes de cambio en la promoción de territorios saludables y sostenibles. Además, la lucha por unas condiciones laborales dignas y el reconocimiento profesional está directamente relacionada con la agenda medioambiental. La sobreexplotación de los trabajadores de la salud refleja el mismo modelo que agota los recursos naturales y compromete la vida en el planeta. Por lo tanto, pensar en la salud planetaria también significa repensar las relaciones laborales y los impactos socioeconómicos que afectan a la enfermería y a la sociedad en su conjunto.

La enfermería tiene la responsabilidad y la oportunidad de estar a la vanguardia en el desarrollo de propuestas y prácticas, promoviendo cuidados que consideren no sólo la salud humana, sino la integridad del planeta. Así, la 86ª SBEn se estructura en torno a tres ejes centrales, que profundizan esta discusión:

**Eje 1. Prácticas de enfermería en múltiples escenarios que promueven la salud del planeta:** destaca el papel de los trabajadores de enfermería en la implementación de prácticas sostenibles, como la gestión responsable de los recursos en los servicios de salud y el abordaje de los impactos del cambio climático en los entornos de atención.

**Eje 2. La educación transformadora como camino para preservar la vida en sus diversas formas:** destaca la importancia de la educación y la alfabetización en la salud planetaria, y los desafíos para la formación y la educación continua. Se aborda también las múltiples perspectivas sobre las diferentes realidades ambientales-territoriales en cuanto a la enseñanza y su potencial transformador y de ampliación del conocimiento y la concienciación, y también sobre el proceso de formación de los profesionales de enfermería con el rol social de agentes de transformación.

**Eje 3. Cambios en el mundo del trabajo y sus impactos en la supervivencia planetaria:** discute la relación entre el trabajo precario, la explotación de los recursos naturales y los desafíos para la valorización de la enfermería, destacando la importancia de las políticas públicas que combatan la sobreexplotación y promuevan condiciones de trabajo decentes.

Al integrar el tema y sus tres ejes, la 86ª SBEn destaca la necesidad de que la enfermería se comprometa en la construcción de un futuro solidario y sostenible, guiado por la ética, la justicia social y el compromiso con la vida en todas sus formas.

# Translation: Saúde planetária: desafios e ação crítica da enfermagem

**DOI:** 10.1590/0034-7167.2025780202pt

**Published:** 2025-05-16

**Authors:** Jacinta de Fatima Sena da Silva

**Affiliations:** I Fiocruz, Brasília. Docente de Programa de Pós-Graduação em Políticas Públicas de Saúde, Angicos-Núcleo de Educação Popular em Saúde e Programa de Saúde, Ambiente e Trabalho. Universidade de Brasília, Departamento de Saúde Coletiva. Pesquisadora e Docente. Presidenta da ABEn (Gestão 2022-2025). Brasília, Distrito Federal, Brasil.

O conceito de saúde planetária^(1)^ abrange a relação entre o bem-estar humano e a preservação dos ecossistemas. Por isso, as intensas mudanças climáticas e a degradação ambiental apresentam desafios para a saúde coletiva que exigem abordagens inovadoras e ações concretas. A enfermagem, historicamente comprometida com o cuidado e a promoção da saúde, tem um papel estratégico nesse processo.

Convergente com esta relevante temática, a Associação Brasileira de Enfermagem (ABEn) propôs para 86ª Semana Brasileira de Enfermagem (SBEn) trazer para o centro do debate um dos temas mais urgentes da atualidade: “Saúde planetária: desafios e ação crítica da enfermagem”.

Nessa perspectiva, a 86ª SBEn busca fortalecer o debate e mobilizar profissionais, estudantes e pesquisadoras(es) para uma agenda positiva baseada na sustentabilidade, equidade e justiça social. Mais do que atender às consequências das emergências ambientais e climáticas, a enfermagem precisa se posicionar ativamente na construção de políticas públicas e na implementação de práticas sustentáveis em seus diversos campos de atuação.

## Debates atuais em iniciativa histórica e celebrativa

A SBEn, realizada anualmente de 12 a 20 de maio, tem como marcos as datas de nascimento de Florence Nightingale e falecimento de Anna Nery. Criada em 1940 pela diretora da Escola de Enfermagem Anna Nery e personagem histórica da ABEn, Laís Netto dos Reys, a SBEn é uma iniciativa da ABEn para fortalecer a enfermagem no Brasil. A partir de 1958, com apoio das seções estaduais, passou a mobilizar escolas e instituições em todo o país. Oficializada em 1960 por Juscelino Kubitschek, a SBEn busca promover encontros científicos, culturais e sociais entre profissionais. A cada ano, a ABEn reafirma esse ato político que se tornou tradição no campo.

A formação e a educação permanente são pilares essenciais para preparar profissionais que compreendam a complexidade dos desafios ambientais, climáticos e sanitários, e sejam agentes de mudança na promoção de territórios saudáveis e sustentáveis. Além disso, a luta por condições dignas de trabalho e reconhecimento profissional se conecta diretamente à pauta ambiental. A super exploração do trabalho em saúde reflete o mesmo modelo que exaure os recursos naturais e compromete a vida no planeta. Dessa forma, pensar em saúde planetária é também repensar as relações de trabalho e os impactos socioeconômicos que afetam a enfermagem e a sociedade como um todo.

A enfermagem tem a responsabilidade e a oportunidade de estar à frente na construção de propostas e práticas, promovendo um cuidado que considere não apenas a saúde humana, mas a integridade do planeta. Assim, a 86ª SBEn estrutura-se em três eixos centrais, que aprofundam essa discussão:

**Eixo 1. Práticas em enfermagem nos múltiplos cenários de atuação que promovem a saúde do planeta:** destaca a atuação das trabalhadoras da enfermagem na implementação de práticas sustentáveis, como a gestão responsável de recursos nos serviços de saúde e o enfrentamento dos impactos das mudanças climáticas nos cenários de cuidado.

**Eixo 2. Educação transformadora como caminho para preservação da vida nas suas diversas formas:** destaca a importância da educação e letramento em saúde planetária, e os desafios para formação e educação permanente. Também aborda os múltiplos olhares para as diversas realidades ambientais-territoriais sobre o ensino e sua potência para a transformação e ampliação do conhecimento e conscientização, e ainda sobre o processo formativo de profissionais de enfermagem com o papel social de agentes de transformação.

**Eixo 3. As mudanças no mundo do trabalho e seus impactos na sobrevivência planetária:** discute a relação entre precarização do trabalho, exploração dos recursos naturais e os desafios para a valorização da enfermagem, ressalstando a importância de políticas públicas que combatam a superexploração e promovam condições dignas de trabalho.

Ao integrar o tema e seus três eixos, a 86ª SBEn ressalta a necessidade de uma enfermagem engajada na construção de um futuro solidário e sustentável, pautado pela ética, justiça social e compromisso com a vida em todas as suas formas.
